# Unraveling a Psychiatric Puzzle: Corticosteroid-Induced Psychosis in Addison’s Disease. A case report

**DOI:** 10.1192/j.eurpsy.2024.1018

**Published:** 2024-08-27

**Authors:** F. Mayor Sanabria, M. Fernández Fariña, M. E. Expósito Durán, C. E. Regueiro Martín-Albo, C. Arroyo Del Val, M. Paz Otero, Í. Alberdi Páramo

**Affiliations:** Instituto de Psiquiatría y Salud Mental, Hospital Clínico San Carlos, Madrid, Spain

## Abstract

**Introduction:**

The spectrum of neuropsychiatric adverse effects of corticosteroids ranges from unspecific symptoms to structured psychotic or affective episodes. We present the case of a 30-year-old woman admitted to our hospital due to behavioral alterations, coinciding with the initiation of treatment with corticosteroid boluses as part of a chemotherapy regimen for gastric adenocarcinoma. She had a previous diagnose of Addison’s disease, undergoing treatment with supplemental corticosteroids.

**Objectives:**

1. To describe the clinical particularities of this case, focusing on the psychopathological aspects and their correlation with the corticoid treatment.
2. To review the available literature regarding the clinical characteristics and management of corticosteroid-induced psychosis, with special interest in patients with adrenal insufficiency that require long term steroid supplementation.

**Methods:**

A review of the patient’s clinical history and complementary tests were carried out. Likewise, we reviewed the available literature in relation to the clinical presentation of corticosteroid-induced psychosis and its pharmacological management.

**Results:**

The patient was admitted to our hospital due to acute behavioural alterations, which temporally coincided with the 4th cycle of FOLFOX chemotherapy and corticosteroid boluses. She presented with incoherent speech, with *non sequitur* answers and glossolalia, as well as dysphoric affect and purposeless behavior. She presented a favorable clinical course after the initiation of treatment with antipsychotics and temporary suspension of corticosteroid treatment.

Manic symptoms are the most common presentation of “corticosteroid-induced psychosis”, with the key characteristic being the temporal association with the corticosteroids administration. Although the discontinuation of steroids generally results in a sudden decrease in symptoms, additional treatment with antipsychotics such as haloperidol or olanzapine might be required for a symptomatic control. In patients with adrenal insufficiency, long-term treatment with lithium or anti-seizure treatments are effective strategies in relapse prevention when a higer steroid dose is required.

**Conclusions:**

- Corticosteroid-induced psychosis is a well described clinical phenomenon, that usually presents with manic symptoms rather than psychotic experiences.
- Progressive discontinuation of corticosteroid treatment usually results in complete cessation of symptoms, but additional psychopharmacological treatment might be required, especially in patients with adrenal insufficiency undergoing long-term corticosteroid treatment.
- This case outlines the psychopathological richness in the presentation of corticosteroid-induced psychosis, and illustrates the challenges in the pharmacological management in patients with adrenal insufficiency.

**Disclosure of Interest:**

None Declared

